# Citation analysis of orthopaedic literature; 18 major orthopaedic journals compared for Impact Factor and SCImago

**DOI:** 10.1186/1471-2474-11-4

**Published:** 2010-01-04

**Authors:** Michiel Siebelt, Teun Siebelt, Peter Pilot, Rolf M Bloem, Mohit Bhandari, Rudolf W Poolman

**Affiliations:** 1 Department of Orthopedic Surgery, Reinier de Graaf Groep, P.O. Box 5501, 2600 GA Delft, The Netherlands; 2 Division of Orthopedic Surgery, McMaster University, Ontario, Canada; 3 Department of Orthopedic Surgery, Onze Lieve Vrouwe Gasthuis, Amsterdam, The Netherlands

## Abstract

**Background:**

One of the disadvantages of the Impact Factor (IF) is self-citation. The SCImago Journal Rank (SJR) indicator excludes self-citations and considers the quality, rather than absolute numbers, of citations of a journal by other journals. The present study re-evaluated the influence of self-citation on the 2007 IF for 18 major orthopaedic journals and investigated the difference in ranking between IF and SJR.

**Methods:**

The journals were analysed for self-citation both overall and divided into a general group (n = 8) and a specialized group (n = 10). Self-cited and self-citing rates, as well as citation densities and IFs corrected for self-citation (cIF), were calculated. The rankings of the 18 journals by IF and by SJR were compared and the absolute difference between these rankings (ΔR) was determined.

**Results:**

Specialized journals had higher self-citing rates (p = 0.01, Δmedian = 9.50, 95%CI -19.42 to 0.42), higher self-cited rates (p = 0.0004, Δmedian = -10.50, 95%CI -15.28 to -5.72) and greater differences between IF and cIF (p = 0.003, Δmedian = 3.50, 95%CI -6.1 to 13.1). There was no significant correlation between self-citing rate and IF for both groups (general: r = 0.46, p = 0.27; specialized: r = 0.21, p = 0.56). When the difference in ranking between IF and SJR was compared between both groups, sub-specialist journals were ranked lower compared to their general counterparts (ΔR: p = 0.006, Δmedian = 2.0, 95%CI -0.39 to 4.39).

**Conclusions:**

Citation analysis shows that specialized orthopaedic journals have specific self-citation tendencies. The correlation between self-cited rate and IF in our sample was large but, due to small sample size, not significant. The SJR excludes self-citations in its calculation and therefore enhances the underestimation in ranking of specialized journals.

## Background

The IF [1] is considered the best reference utensil for evaluation of scientific journals although its limitations have already been described extensively [2-7]. Major points of criticism are the lack of quality assessment for citations [5], poor comparability between different domains of interest per journal [6] and the mainly English language in publications. A major problem with the IF for journals is self-citation, defined in Journal Citation Reports as referring to articles from the same journal [1]. Due to these limitations and the simplicity of calculating the IF, it would be relatively easy for editors to manipulate it. A journal's IF can be increased artificially by using self-citations, publishing relatively many review articles and limiting the number of articles included.

The internet search engine Google™ uses a PageRank algorithm to determine page ranking after a specific search query. Several authors have advocated a similar algorithm for the evaluation of scientific journals. Falagas et al. [5] recently described the application of a PageRank algorithm to the Scopus database to produce the SCImago Journal Rank (SJR) indicator. The SJR represents awarded prestige per article in the analysed year and is calculated using a complicated iterative formula. Self-citations do not contribute to the SJR, since a journal can receive prestige only from other journals, not from itself [8]. The SJR has several other benefits [5], such as the greater number of journals and languages included in its database and the fact that it is open-source software (free of charge).

In the present study we first aimed to evaluate the influence of self-citations on the 2007 IF for 18 major orthopaedic journals, including sub-specialist journals. These journals publish specialized articles concerning a narrow field of interest and as such are expected to receive relatively few citations from other journals. We therefore hypothesized that self-citation rates are higher for specialized journals than for the general orthopaedic literature. Secondly, we investigated ranking of orthopaedic journals with the SJR. This indicator excludes self-citations, therefore we hypothesized that the SJR ranks specialized journals comparatively lower than does the IF.

## Methods

We included and analyzed 18 orthopaedic journals, making a distinction between general and specialized orthopaedic journals. Two orthopaedic clinicians participating in this study individually allocated journals to either the general or the specialized group.

The general group included the following journals: *Acta Orthopaedica (Acta)*, *Archives of Orthopaedics and Trauma (AOTS), BMC Musculoskeleletal Disorders (BMCMD)*, *Clinical Orthopaedics and Related Research (CORR)*, *International Orthopaedics (Int Orthop), the American Volume of Journal of Bone and Joint Surgery (JBJS [Am])*, *the British volume of Journal of Bone and Joint Surgery (JBJS [Br]) *and *Orthopaedic Clinics of North America (OCNA)*.

The specialized orthopaedic journals included in the study were: *American Journal of Sports Medicine (AJSM)*, *Arthroscopy*, *European Spine Journal (ESJ)*, *Foot and Ankle International (FAI), Journal of Arthroplasty (JOA)*, *Journal of Orthopaedic Trauma (JOT)*, *Journal of Pediatric Orthopaedics (JPO)*, *Journal of shoulder and elbow surgery (JSES)*, *Knee surgery sports traumatology arthroscopy (KSSTA) *and *Spine*.

For both groups self-citation was analysed for the 2007 IF. The self-citing rate, self-cited rate and citation density, parameters known to influence the IF [2], were calculated for each of these journals as defined by the ISI Web of Science [1]. Also, all IF were corrected for the influence of self-citations (cIF) [1]; for individual journal evaluation, an absolute change of >0.5 in IF (ΔIF = IF - cIF) was considered substantial. To estimate the influence of self-citation on the IF, journals in both groups were correlated for self-citing rate and IF.

The ***self-citing rate ***[1] was calculated by dividing the number of self-cited articles in journal X in 2007 by the total amount of citations by that journal in 2007. For example, in 2007 *AJSM *contained 1542 citations of *AJSM *articles on a total of 8089 citations; the self-citing rate was 1542/8089 = 19% (Table 1).

**Table 1:** Self-citing rate, self-cited rate, citation density and IF 2007

Journal*	Self-citing^a^(%)	Self-cited^b^(%)	Citation density (%)	Impact Factor 2007	Corrected IF (cIF)	Difference^c^(ΔIF = IF -- cIF)
**General orthopaedic journals**						

*Acta*	7	5	31	1.285	1.122	0.163

*AOTS*	2	4	23	0.913	0.873	0.040

*BMC MD*	1	6	38	1.323	1,226	0.097

*CORR*	15	8	31	1.891	1.626	0.265

*Int Orthop*	4	8	22	0.903	0.796	0.107

*JBJS [Am]*	7	6	36	2.487	2.363	0.124

*JBJS [Br]*	6	7	28	1.868	1.664	0.204

*OCNA*	1	1	53	1.692	1.692	0.000

**Specialized orthopaedic journals**						

*AJSM*	19	14	34	3.397	2.731	0.666

*Arthroscopy*	27	23	22	2.296	1.550	0.746

*ESJ*	5	17	34	2.021	1.545	0.476

*FAI*	24	38	23	0.956	0.581	0.375

*JoA*	14	15	21	1.609	1.403	0.206

*JOT*	11	13	29	1.429	1.199	0.230

*JPO*	15	9	34	1.036	0.927	0.109

*JSES*	14	21	24	1.348	1.158	0.190

*KSSTA*	5	16	26	1.626	1.314	0.312

*Spine*	29	20	32	2.499	1.871	0.628

*Acta Orthopaedica (Acta)*, *American Journal of Sports Medicine (AJSM)*, *Arthroscopy*, *Archives of Orthopaedics and Trauma (AOTS), BMC Musculoskeleletal Disorders (BMC MD), Clinical Orthopaedics and Related Research (CORR)*, *European Spine Journal (ESJ)*, *Foot and Ankle International (FAI), International Orthopaedics (Int Orthop), Journal of Arthroplasty (JOA)*, *the American Volume of Journal of Bone and Joint Surgery (JBJS [Am])*, *the British volume of Journal of Bone and Joint Surgery (JBJS [Br])*, *Journal of Orthopaedic Trauma (JOT)*, *Journal of Pediatric Orthopaedics (JPO)*, *Journal of shoulder and elbow surgery (JSES)*, *Knee surgery sports traumatology arthroscopy (KSSTA)*, *Orthopaedic Clinics of North America (OCNA)*, and *Spine*.

^a ^p = 0.01, Mann--Whitney between general and specialized journals.

^b^p = 0.0004, Mann--Whitney between general and specialized journals.

^c ^p = 0.003, Mann--Whitney between general and specialized journals.

The ratio between the number of self-cited articles in journal X in 2007 and the total amount of citations received in 2007 for articles in journal X is the ***self-cited rate ***[1]. For example, *AJSM *was cited 10,711 times in total, of which 1542 by itself; the self-cited rate was 1542/10711 = 14% (Table 1).

The ***citation density ***[1] was determined by dividing the total number of references in journal X in 2007 by the total number of articles published in that journal in 2007. For example, the 239 articles published in *AJSM *in 2007 contained a total of 8089 references; the citation density was 8089/239 = 34 citations per article (Table 1).

We retrieved information regarding ranking by SJR via the SCImago journal- and country-rank website developed by the SCImago research group [8]; the journals were selected by matching international standard serial number (ISSN) found in the JCR. Since the IF ranks 6426 journals and the SJR 15,922, absolute rankings by IF and SJR are not comparable. Therefore only the 18 journals included in our analysis were mutually ranked for IF (R_IF_) and SJR (R_SJR_). The difference between R_IF _and R_SJR_, expressed as ΔR, was calculated to check for a possible difference between both rankings.

### Statistical analysis

Statistical analysis was performed using Prism v5.00 for Windows (Graphpad Software Inc, San Diego, CA, USA). Analysing differences between general and specialized journals for the IF, the following specifics were compared using a Mann-Whitney test: self-citing rate, self-cited rate, citation density, ΔIF, R_IF_, R_SJR _and ΔR. We determined the medians, the first- and third-quartile values, and the difference between the medians (Δmedian = median_general _- median_specialized_) and their confidence intervals. A Spearman rank correlation coefficient was computed to estimate the correlation between self-citing rates and IF, as well as between the journal rankings by IF and by SJR. For all tests, p values < 0.05 were considered significant.

## Results

### Self-citation rates

The journals with the highest self-citing rates were *CORR *in the general group (15%) and *Spine *in the specialized group (29%) (Table 1); the lowest rates were for *BMC MD/OCNA *(general group, 1%) and *KSSTA/ESJ *(specialized group, 5%). The highest self-cited rates were for *CORR/Int Orthop *(general, 14%) and *FAI *(specialized, 38%), the lowest rates for *OCNA *(1%) and *JPO *(9%). *Arthroscopy *(0.75), *AJSM *(0.67) and *Spine *(0.63), all experience substantial declines in IF when corrected for self-citation (cIF).

**Figure 1:**
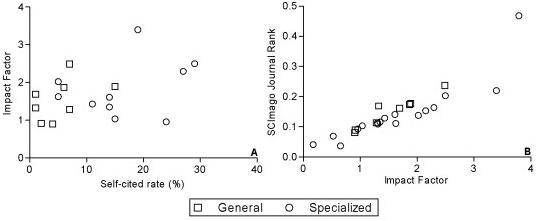
**Correlation Analysis. **A: Correlation between self-cited rate (%) and impact factor B: Correlation between IF and SJR

Table 2 shows the medians and first- and third-quartile values of the citation analysis. Specialized journals have significantly higher self-citing rates (Δmedian = 9.50, 95%CI -19.42 to 0.42, p = 0.01) and self-cited rates (Δmedian = -10.50, 95%CI -15.28 to -5.72, p = 0.0004). Both types of journal tend to use similar numbers of citations per published article (Δmedian = 3.50; 95%CI -6.1 to 13.1, p = 0.35). The absolute difference between IF and cIF proved to be greater for specialized journals (Δmedian = -0.22; 95%CI -0.45 to -0.01; p = 0.003). There was no significant correlation between the self-cited rate and the IF for either the general (r = 0.46; p = 0.27) or the specialized group (r = 0.21; p = 0.56) (figure 1A).

**Table 2:** Citation analysis for general and specialized journal groups

	General (n = 8)			Specialized (n = 10)		
	
	1st Quartile	Median	3rd Quartile	1st Quartile	Median	3rd Quartile
	
Self-citing rate	1.25	5.00	7.00	9.50	14.50	24.75
Self-cited rate	4.25	6.00	7.75	13.75	16.50	21.50

Citation density	24.25	31.00	37.50	22.75	27.50	34.00

ΔIF	0.05	0.12	0.19	0.20	0.34	0.64

### Comparative Ranking: IF versus SCImago (Table 3)

The rankings by IF and SJR for the 18 journals in our analysis are presented in Table 3. For the SJR, an orthopaedic ranking is possible for the sixteen journals represented in the subcategory *Medicine: Orthopaedics and Sport Medicine *in the SJR database, but not for *BMC MD *(included in the subcategory *Medicine: Miscellaneous*) and *Spine *(included in the subcategory *Biochemistry, Genetics and Molecular Biology*).

**Table 3:** Comparative rankings of orthopaedic journals by journal impact factor and SCImago journal rank indicator

Journal Impact Factor					SCImago journal rank indicator			
**Study rank** **(n = 18)**	**Total rank** **(n = 6426)**	**Orthopaedic rank** **(n = 48)**	**Value**	**Journal**	**Value**	**Orthopaedic rank** **(n = 120)**	**Total rank** **(n = 15922)**	**Study rank** **(n = 18)**

**1**	785	2	3.397	*AJSM	0.220	8	788	**2**

**2**	1323	3	2.499	*Spine	0.203	-	-	**3**

**3**	1330	4	2.487	JBJS [Am]	0.237	7	724	**1**

**4**	1514	6	2.296	*Arthroscopy	0.164	13	1080	**7**

**5**	1818	10	2.021	*ESJ	0.138	22	1281	**10**

**6**	1999	11	1.891	CORR	0.177	11	994	**4**

**7**	2023	12	1.868	JBJS [Br]	0.174	12	1017	**5**

**8**	2280	13	1.692	OCNA	0.162	15	1101	**8**

**9**	2376	16	1.626	*KSSTA	0.111	34	1589	**14**

**10**	2406	17	1.609	*JoA	0.141	21	1246	**9**

**11**	2719	20	1.429	*JOT	0.129	24	1364	**11**

**12**	2868	21	1.348	*JSES	0.116	29	1515	**12**

**13**	2907	22	1.323	BMC MD	0.169	-	2907	**6**

**14**	2996	25	1.285	Acta	0.113	31	1540	**13**

**15**	3541	28	1.036	*JPO	0.103	40	1679	**15**

**16**	3757	29	0.956	*FAI	0.093	44	1792	**16**

**17**	3856	30	0.913	AOTS	0.089	45	1838	**17**

**18**	3882	31	0.903	Int Orthop	0.081	50	1965	**18**

								

**3**	1330	4	2.487	JBJS [Am]	0.237	7	1684	**1**

**1**	785	2	3.397	*AJSM	0.220	8	1815	**2**

**2**	1323	3	2.499	*Spine	0.203	-	1954	**3**

**6**	1999	11	1.891	CORR	0.177	11	2217	**4**

**7**	2023	12	1.868	JBJS [Br]	0.174	12	2258	**5**

**13**	2907	22	1.323	BMC MD	0.169	-	2907	**6**

**4**	1514	6	2.296	*Arthroscopy	0.164	13	2394	**7**

**8**	2280	13	1.692	OCNA	0.162	15	2431	**8**

**10**	2406	17	1.609	*JoA	0.141	21	2743	**9**

**5**	1818	10	2.021	*ESJ	0.138	22	2802	**10**

**11**	2719	20	1.429	*JOT	0.129	24	2985	**11**

**12**	2868	21	1.348	*JSES	0.116	29	3284	**12**

**14**	2996	25	1.285	Acta	0.113	31	3338	**13**

**9**	2376	16	1.626	*KSSTA	0.111	34	3432	**14**

**15**	3541	28	1.036	*JPO	0.103	40	3655	**15**

**16**	3757	29	0.956	*FAI	0.093	44	3979	**16**

**17**	3856	30	0.913	AOTS	0.089	45	4121	**17**

**18**	3882	31	0.903	Int Orthop	0.081	50	4459	**18**

*Acta Orthopaedica (Acta)*, *American Journal of Sports Medicine (AJSM)*, *Arthroscopy*, *Archives of Orthopaedics and Trauma (AOTS), BMC Musculoskeleletal Disorders (BMC MD), Clinical Orthopaedics and Related Research (CORR)*, *European Spine Journal (ESJ)*, *Foot and Ankle International (FAI), International Orthopaedics (Int Orthop), Journal of Arthroplasty (JOA)*, *the American Volume of Journal of Bone and Joint Surgery (JBJS [Am])*, *the British volume of Journal of Bone and Joint Surgery (JBJS [Br])*, *Journal of Orthopaedic Trauma (JOT)*, *Journal of Pediatric Orthopaedics (JPO)*, *Journal of shoulder and elbow surgery (JSES)*, *Knee surgery sports traumatology arthroscopy (KSSTA)*, *Orthopaedic Clinics of North America (OCNA)*, and *Spine*.

*: Specialized journals are marked with an asterisk.

a: Orthopaedic rankings for BMC MD and Spine are not available, as they are not in the same sub-category as the other 16 journals.

In comparing SJR rank relative to IF rank, seven journals maintained their rank (*OCNA, JOT, JSES, JPO, FAI, AOTS, Int Orthop*), six improved their rank (*JBJS [Am], CORR, JBJS [Br], JoA, Acta, BMC MD*) and five experienced a decline in rank (*AJSM, Spine, Arthroscopy, ESJ, KSSTA*). The greatest differences between SJR and IF rank were seen for *BMC MD *(seven places up in SJR) and *ESJ/KSSTA *(four places down). Despite these changes, a strong correlation was found between IF and SJR (general: r = 0,98, p < 0.0001) (specialized: r = 0.93, p < 0.0001) (figure 1B).

Table 4 presents the differences in ranking between both groups, IF (Δmedian = 1.00, 95%CI -7.13 to 9.13, p = 0.41) and for SJR (Δmedian = -3.50, 95%CI -12.2 to 5.2, p = 0.76). There was a significant difference in ΔR between both groups (Δmedian = 2.0, 95%CI -0.39 to 4.39, p = 0.006).

**Table 4:** Comparison of IF and SJR rankings

	General (n = 8)			Specialized (n = 10)		
	
	**1**^st^**Quartile**	Median	**3**^rd^**Quartile**	**1**^st^**Quartile**	Median	**3**^rd^**Quartile**
R_IF_	6.25	10.50	16.25	3.50	9.50	12.75

R_SJR_	4.25	7.00	16.00	6.00	10.50	14.25

ΔR = R_IF _- R_SJR_	0.0	1.50	2.00	-3.50	-0.50	0.0

## Discussion

### Key findings

Our study revealed the following: (1) Specialized journals receive proportionally more self-citations with a strong influence on the IF. (2) The SJR shows a strong relation with the IF. (3) Correcting for self-citation with the newly introduced SJR results in substantial individual changes for journal ranking. (4) Specialized journals tend to drop, whereas general journals will climb, in rank when the SJR is applied instead of IF.

Group-specific characteristics were clearly visible. Sub-specialist journals have higher self-citing (p = 0.01) and self-cited rates (p = 0.0004), also expressed in a greater difference between IF and corrected IF for self-citations (p = 0.003). Despite the elaborate iterative calculation of the SJR, there exists a very strong correlation between IF and SJR ranking (general: r = 0.98, p < 0.0001) (specialized: r = 0.93, p < 0.0001). Comparing the mutual difference in ranking for SJR and IF between both groups showed an increased contrast (p = 0.003).

### Strength and weaknesses of our study

Our study is strengthened by the analysis of two separate groups of orthopaedic literature: general and specialized orthopaedic journals. Hakkalamani et al. [9] excluded several journals because of a suspected increased self-citation because of subject subspecialty. Applying standard definitions for self-citing rate, self-cited rate and citation index from the ISI Web of Science to these journals, we were able to identify specific citation patterns for specialized journals. Comparison of the Impact Factor with the newly introduced SCImago journal rank indicator elucidated whether citation analysis based on a PageRank algorithm provides improved quality assessment of orthopaedic literature over journal indicators.

A limitation of this study is the evaluation of journal citations for 2007 only, and not for other years of publication. Furthermore, the distinction made between general and specialized journals is arbitrary; however, distinguishing journal categories by domain of interest is plausible and is coherent with work published previously [9]. Therefore, we believe that applying widely accepted methods for citation analysis to our analysis groups generated reliable data.

### Previous Literature

Self-citation indicators that affect the IF are not limited to the orthopaedic literature [9]. Similar relations have been demonstrated for anaesthesia [10] and radiology [11]. The high self-citation indexes found within these specialized fields of medical science prompted suggestions that self-citations should be eliminated from the calculation of the IF. However for the 2007 IF in orthopaedic literature we did not find a similar distinct influence of self-citations. The high self-citation indexes for specialized journals relative to general journals rather indicate that these journals serve a small and isolated field within the orthopaedic literature [10]. The question can be posed whether high self-citation rates within a specialized domain of interest are indications for low-quality publications or rather reflect overall high quality? Papers published in these specialized journals are likely to have a high impact in their field of interest [6].

### Implications of our study

Postma has stated that the impact of evolutionary papers published in multidisciplinary journals is substantially overestimated by their overall impact factor and, on the other hand, that the impact of papers in more specialized journals is significantly underestimated [6]. We agree that the IF has evolved for many users into an indicator of quality for articles, but one should always be aware of the fact that article citation rates determine the journal impact factor, not vice versa [3]. The differences found in our analysis of two groups of orthopaedic journals do not represent a difference in quality of articles published in these journals. They confirm that these groups publish different types of research, that is, either multidisciplinary or sub-specialized orthopaedic literature.

The SJR, based on an PageRank algorithm, provides a more sophisticated alternative for the IF and eliminates the effect of self-citations, which might be desirable if self-citation is seen as a negative aspect. Our analysis showed that the SJR bears a high resemblance with the IF, but further enhances differences between general and specialized literature. As a result of the iterative calculation of the SJR, the contrast between both groups of literature will increase over time.

## Conclusions

Even though there was no significant correlation between self-cited rate and IF, there is a strong relation between these both indices. Self-citation indexes tended to be higher for specialized orthopaedic journals. The SJR corrects for self-citations, which increases the gap in overall ranking between specialized and general journals, this reflects a difference in field of interest rather than quality.

## List of abbreviations

IF: Impact Factor; SJR: SCImago Journal Rank Indicator; Acta: Acta Orthopaedica; AJSM: American Journal of Sports Medicine; AOTS: Archives of Orthopaedics and Trauma; BMCMD: BMC Musculoskeleletal Disorders; CORR: Clinical Orthopaedics and Related Research; ESJ: European Spine Journal; FAI: Foot and Ankle International; Int Orthop: International Orthopaedics; JBJS [Am]: the American Volume of Journal of Bone and Joint Surgery; JBJS [Br]: the British volume of Journal of Bone and Joint Surgery; JOA: Journal of Arthroplasty; JOT: Journal of Orthopaedic Trauma; JPO: Journal of Pediatric Orthopaedics; JSES: Journal of shoulder and elbow surgery; KSSTA: Knee surgery sports traumatology arthroscopy; R_IF_: IF ranking; R_SJR_: SJR ranking; ΔR: difference between IF and SJR ranking

## Competing interests

The authors declare that they have no competing interests.

## Authors' contributions

MS: study design; acquisition, analysis and interpretation of data; statistical analysis; drafting and revising of the manuscript, read and approved final manuscript. TS: study design; acquisition, analysis and interpretation of data; statistical analysis; drafting and revising of the manuscript, read and approved final manuscript. PP: study design, data interpretation; revising of the manuscript, read and approved final manuscript. RMB: study design, data interpretation; revising of the manuscript, read and approved final manuscript. MB: study design, data interpretation; revising of the manuscript, read and approved final manuscript. RWP: study design, data interpretation; revising of the manuscript, read and approved final manuscript.

## Pre-publication history

The pre-publication history for this paper can be accessed here:

http://www.biomedcentral.com/1471-2474/11/4/prepub
